# Bis[μ-*N*-(pyridin-2-ylmeth­yl)pyridin-3-amine-κ^2^ *N*:*N*′]disilver(I) bis­(perchlorate) dimethyl sulfoxide disolvate

**DOI:** 10.1107/S1600536813026585

**Published:** 2013-10-02

**Authors:** Suk-Hee Moon, Ki-Min Park

**Affiliations:** a Department of Food & Nutrition, Kyungnam College of Information and Technology, Busan 617-701, Republic of Korea; b Department of Chemistry and Research Institute of Natural Sciences, Gyeongsang National University, Jinju 660-701, Republic of Korea

## Abstract

In the binuclear title compound, [Ag_2_(C_11_H_11_N_3_)_2_](ClO_4_)_2_·2C_2_H_6_SO, the complex cation is centrosymmetric, with the unique Ag^I^ cation coordinated by two pyridine N atoms from two symmetry-related *N*-(pyridine-2-ylmeth­yl)pyridine-3-amine ligands in a geometry slightly distorted from linear [N—Ag—N = 170.78 (9)°], resulting in the formation of a 16-membered cyclic dimer. The two pyridine rings coordinating to the Ag^I^ atom are almost perpendicular to each other [dihedral angle = 87.73 (10)°]. Inter­molecular Ag⋯O inter­actions [3.149 (3) and 2.686 (3) Å], N—H⋯O and C—H⋯O hydrogen bonds and C—H⋯π inter­actions between the cyclic dimers and the anions or the solvent mol­ecules lead to the formation of a three-dimensional supra­molecular network.

## Related literature

For structures of Ag^I^ coordination polymers with symmetrical dipyridyl ligands, see: Lee *et al.* (2012 ▶); Leong & Vittal (2011 ▶); Park *et al.* (2010 ▶) and of Ag^I^ coordination polymers with unsymmetrical dipyridyl ligands, see: Moon & Park (2013 ▶); Zhang *et al.* (2013 ▶). For the synthesis of the ligand, see: Foxon *et al.* (2002 ▶); Lee *et al.* (2008 ▶).
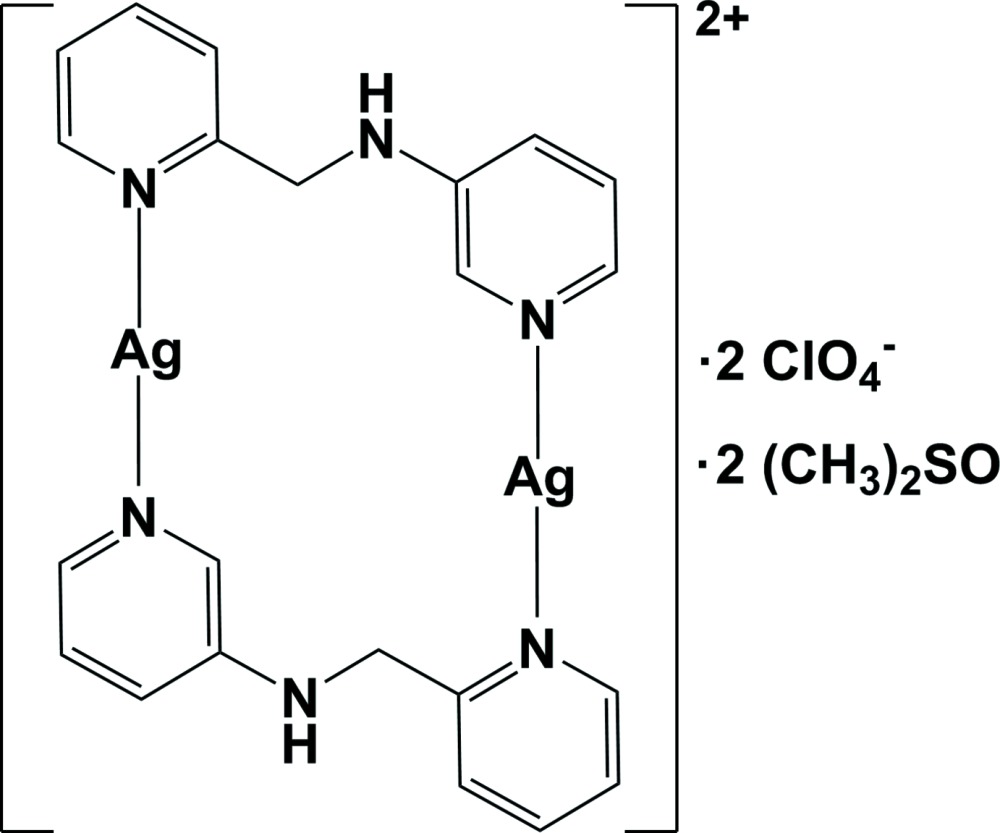


## Experimental

### 

#### Crystal data

[Ag_2_(C_11_H_11_N_3_)_2_](ClO_4_)_2_·2C_2_H_6_OS
*M* *_r_* = 941.35
Monoclinic, 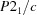

*a* = 7.3620 (3) Å
*b* = 11.1227 (5) Å
*c* = 21.1248 (10) Å
β = 95.328 (1)°
*V* = 1722.34 (13) Å^3^
*Z* = 2
Mo *K*α radiation
μ = 1.48 mm^−1^
*T* = 173 K
0.45 × 0.20 × 0.20 mm

#### Data collection

Bruker SMART CCD area-detector diffractometer
9537 measured reflections
3367 independent reflections
3096 reflections with *I* > 2σ(*I*)
*R*_int_ = 0.049

#### Refinement

*R*[*F* ^2^ > 2σ(*F* ^2^)] = 0.033
*wR*(*F* ^2^) = 0.086
*S* = 1.02
3367 reflections
217 parameters
H-atom parameters constrained
Δρ_max_ = 0.61 e Å^−3^
Δρ_min_ = −0.65 e Å^−3^

### 

Data collection: *SMART* (Bruker, 2000 ▶); cell refinement: *SAINT-Plus* (Bruker, 2000 ▶); data reduction: *SAINT-Plus*; program(s) used to solve structure: *SHELXS97* (Sheldrick, 2008 ▶); program(s) used to refine structure: *SHELXL97* (Sheldrick, 2008 ▶); molecular graphics: *DIAMOND* (Brandenburg, 2005 ▶); software used to prepare material for publication: *SHELXTL* (Sheldrick, 2008 ▶).

## Supplementary Material

Crystal structure: contains datablock(s) I, New_Global_Publ_Block. DOI: 10.1107/S1600536813026585/sj5353sup1.cif

Structure factors: contains datablock(s) I. DOI: 10.1107/S1600536813026585/sj5353Isup2.hkl

Additional supplementary materials:  crystallographic information; 3D view; checkCIF report

## Figures and Tables

**Table 1:** Hydrogen-bond geometry (Å, °) *Cg* is the centroid of the N2/C7–C11 pyridine ring.

*D*—H⋯*A*	*D*—H	H⋯*A*	*D*⋯*A*	*D*—H⋯*A*
N3—H3⋯O1	0.88	2.32	3.081 (4)	144
C1—H1⋯O2^i^	0.95	2.56	3.214 (4)	126
C6—H6*A*⋯O5^ii^	0.99	2.55	3.495 (4)	160
C11—H11⋯O5^iii^	0.95	2.55	3.191 (4)	125
C12—H12*C*⋯O4^iv^	0.98	2.49	3.270 (5)	137
C13—H13*A*⋯*Cg*	0.98	3.36	4.116 (5)	136

Symmetry codes: (i) 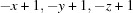; (ii) 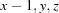; (iii) 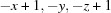; (iv) 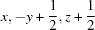.

**Fig. 1.**
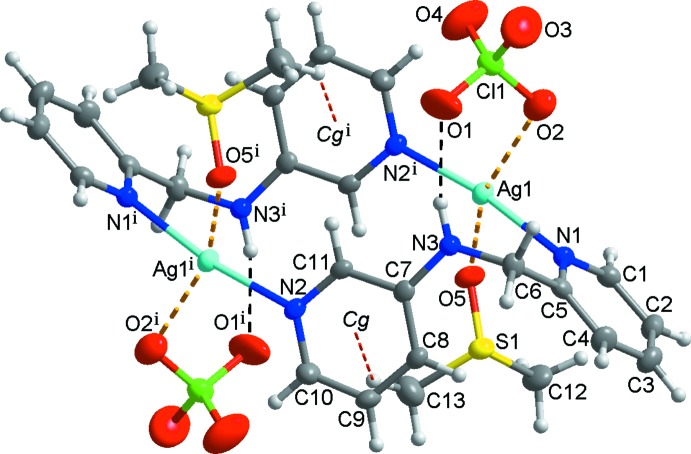
A view of the molecular structure of the title compound, with displacement ellipsoids drawn at the 50% probability level. Intermolecular Ag···O interactions, N–H···O hydrogen bonds and C–H···π interactions are shown as yellow, black and red dashed lines, respectively. [Symmetry code: (i) 1 - x, - y, 1 - z].

**Fig. 2.**
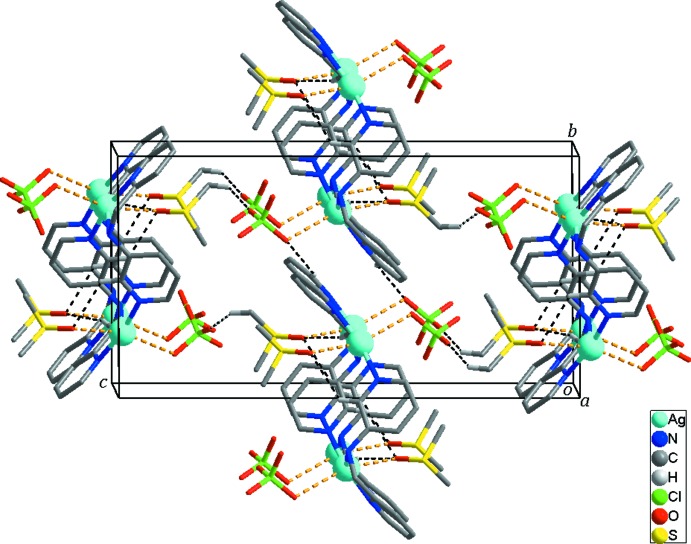
The three-dimensional supramolecular structure formed through intermolecular Ag···O (yellow dashed lines) and C–H···O interactions (black dashed lines).

## supplementary crystallographic information

### 1. Comment

In the development of silver(I) coordination polymers with fascinating structures, numerous symmetrical dipyridyl ligands with nitrogen donor atoms in the same positions on the two terminal pyridines are employed due to their easy synthesis (Lee *et al.*, 2012; Leong & Vittal, 2011; Park *et al.*, 2010). Despite the rapid growth in the Ag^I^ coordination chemistry, however, the investigation of Ag^I^ coordination polymers using unsymmetrical dipyridyl ligands with nitrogen donor atoms on different positions in the two terminal pyridines still remains lacking (Moon *et al.*, 2013; Zhang *et al.*, 2013). Herein, we report the crystal structure of the title compound prepared by the reaction of silver(I) perchlorate with the unsymmetrical dipyridyl ligand *N*-(pyridine-3-ylmethyl)pyridine-2-amine. This was synthesized by the reaction of 2-aminopyridine and 3-pyridinecarboxaldehyde according to literature procedures (Foxon *et al.*, 2002; Lee *et al.*, 2008).

The binuclear cation of the title compound, [Ag_2_(C_11_H_11_N_3_)_2_](ClO_4_)_2_(C_2_H_6_SO)_2_, is located on an inversion centre. Therefore, the asymmetric unit of the compound consists of an Ag^I^ cation, an *N*-(pyridine-3-ylmethyl)pyridine-2-amine ligand, a perchlorate anion and a molecule of dimethyl sulfoxide (DMSO) solvent. The two Ag^I^ centres, each in a geometry slightly distorted from linear [N–Ag–N 170.78 (9)°], are coordinated by two pyridine N atoms from the two symmetry-related *N*-(pyridine-3-ylmethyl)pyridine-2-amine ligands, leading to the formation of a centrosymmetric 16-membered cyclic dimer (Fig. 1). The two pyridine rings coordinated to each Ag^I^ centre are almost perpendicular to each other [dihedral angle = 87.73 (10)°]. Moreover, each Ag^I^ centre in the cyclic dimer interacts weakly with the N atom of a secondary amine group [Ag1···N3 2.699 (3) Å] and the O atom of a DMSO solvent molecule and a ClO_4_^-^ anion [Ag1···O5 2.686 (3) Å and Ag1···O2 3.149 (3) Å] (Fig. 1).

In the crystal structure, the cyclic units interact with the ClO_4_^-^ anions and the DMSO solvent molecules *via* intermolecular N–H···O and C–H···O hydrogen-bonds and C–H···π interactions (Table 1, Fig. 1). These interactions lead to the construction of a three-dimensional supramolecular network (Fig. 2).

### 2. Experimental

The ligand (*N*-(pyridin-3-ylmethyl)pyridine-2-amine) was prepared according to a procedure described by Foxon *et al.* (2002) and Lee *et al.* (2008). Crystals of the title compound suitable for X-ray analysis were obtained by vapor diffusion of diethyl ether into a DMSO solution of the white precipitate afforded by the reaction of the ligand with silver(I) perchlorate in the malar ratio 1:1 in methanol.

### 3. Refinement

All H atoms were positioned geometrically and refined using a riding model, with d(C—H) = 0.95 Å for C*sp*^2^–H, 0.88 Å for amine N–H, 0.98 Å for methyl C–H and 0.99 Å for methylene C–H. For all H atoms *U*_iso_(H) = 1.2*U*_eq_(C,N).

### Crystal data

**Table:**

[Ag_2_(C_11_H_11_N_3_)_2_](ClO_4_)_2_·2C_2_H_6_OS	*F*(000) = 944
*M**_r_* = 941.35	*D*_x_ = 1.815 Mg m^−^^3^
Monoclinic, *P*2_1_/*c*	Mo *K*α radiation, λ = 0.71073 Å
Hall symbol: -P 2ybc	Cell parameters from 7360 reflections
*a* = 7.3620 (3) Å	θ = 2.7–28.3°
*b* = 11.1227 (5) Å	µ = 1.48 mm^−^^1^
*c* = 21.1248 (10) Å	*T* = 173 K
β = 95.328 (1)°	Block, colorless
*V* = 1722.34 (13) Å^3^	0.45 × 0.20 × 0.20 mm
*Z* = 2	

### Data collection

**Table:**

Bruker SMART CCD area-detector diffractometer	3096 reflections with *I* > 2σ(*I*)
Radiation source: fine-focus sealed tube	*R*_int_ = 0.049
Graphite monochromator	θ_max_ = 26.0°, θ_min_ = 1.9°
φ and ω scans	*h* = −9→8
9537 measured reflections	*k* = −13→13
3367 independent reflections	*l* = −25→18

### Refinement

**Table:**

Refinement on *F*^2^	Primary atom site location: structure-invariant direct methods
Least-squares matrix: full	Secondary atom site location: difference Fourier map
*R*[*F*^2^ > 2σ(*F*^2^)] = 0.033	Hydrogen site location: inferred from neighbouring sites
*wR*(*F*^2^) = 0.086	H-atom parameters constrained
*S* = 1.02	*w* = 1/[σ^2^(*F*_o_^2^) + (0.0399*P*)^2^ + 3.5438*P*] where *P* = (*F*_o_^2^ + 2*F*_c_^2^)/3
3367 reflections	(Δ/σ)_max_ = 0.001
217 parameters	Δρ_max_ = 0.61 e Å^−^^3^
0 restraints	Δρ_min_ = −0.65 e Å^−^^3^

### Special details

**Table:**

Geometry. All e.s.d.'s (except the e.s.d. in the dihedral angle between two l.s. planes) are estimated using the full covariance matrix. The cell e.s.d.'s are taken into account individually in the estimation of e.s.d.'s in distances, angles and torsion angles; correlations between e.s.d.'s in cell parameters are only used when they are defined by crystal symmetry. An approximate (isotropic) treatment of cell e.s.d.'s is used for estimating e.s.d.'s involving l.s. planes.
Refinement. Refinement of *F*^2^ against ALL reflections. The weighted *R*-factor *wR* and goodness of fit *S* are based on *F*^2^, conventional *R*-factors *R* are based on *F*, with *F* set to zero for negative *F*^2^. The threshold expression of *F*^2^ > σ(*F*^2^) is used only for calculating *R*-factors(gt) *etc*. and is not relevant to the choice of reflections for refinement. *R*-factors based on *F*^2^ are statistically about twice as large as those based on *F*, and *R*- factors based on ALL data will be even larger.

### Fractional atomic coordinates and isotropic or equivalent isotropic displacement parameters (Å^2^)

**Table:**

	*x*	*y*	*z*	*U*_iso_*/*U*_eq_	
Ag1	0.50109 (3)	0.25805 (2)	0.484406 (11)	0.02344 (10)	
N1	0.3332 (3)	0.3841 (2)	0.53484 (12)	0.0200 (5)	
N2	0.3266 (3)	−0.1183 (2)	0.55086 (12)	0.0200 (5)	
N3	0.1611 (3)	0.1670 (2)	0.48358 (12)	0.0224 (5)	
H3	0.2056	0.1604	0.4465	0.027*	
C1	0.4183 (4)	0.4681 (3)	0.57264 (14)	0.0229 (6)	
H1	0.5437	0.4832	0.5686	0.028*	
C2	0.3334 (4)	0.5334 (3)	0.61688 (15)	0.0269 (7)	
H2	0.3984	0.5924	0.6424	0.032*	
C3	0.1510 (5)	0.5108 (3)	0.62316 (16)	0.0305 (7)	
H3A	0.0886	0.5533	0.6536	0.037*	
C4	0.0616 (4)	0.4257 (3)	0.58450 (17)	0.0288 (7)	
H4	−0.0639	0.4096	0.5879	0.035*	
C5	0.1546 (4)	0.3632 (3)	0.54062 (14)	0.0201 (6)	
C6	0.0546 (4)	0.2732 (3)	0.49703 (16)	0.0240 (6)	
H6A	−0.0562	0.2469	0.5164	0.029*	
H6B	0.0145	0.3135	0.4563	0.029*	
C7	0.1930 (4)	0.0766 (3)	0.52826 (14)	0.0186 (6)	
C8	0.1501 (4)	0.0853 (3)	0.59090 (15)	0.0231 (6)	
H8	0.0907	0.1548	0.6051	0.028*	
C9	0.1955 (5)	−0.0089 (3)	0.63220 (15)	0.0275 (7)	
H9	0.1657	−0.0049	0.6750	0.033*	
C10	0.2841 (4)	−0.1091 (3)	0.61127 (15)	0.0265 (7)	
H10	0.3156	−0.1727	0.6402	0.032*	
C11	0.2807 (4)	−0.0285 (3)	0.51076 (14)	0.0187 (6)	
H11	0.3090	−0.0362	0.4680	0.022*	
Cl1	0.22314 (10)	0.27926 (7)	0.31521 (4)	0.02713 (18)	
O1	0.1356 (6)	0.1764 (3)	0.33725 (17)	0.0789 (13)	
O2	0.2542 (4)	0.3637 (3)	0.36785 (14)	0.0448 (7)	
O3	0.1082 (5)	0.3355 (4)	0.26650 (16)	0.0698 (10)	
O4	0.3925 (5)	0.2503 (3)	0.2927 (2)	0.0716 (12)	
S1	0.60059 (11)	0.23249 (7)	0.65444 (4)	0.02617 (18)	
O5	0.7044 (3)	0.2308 (2)	0.59577 (12)	0.0329 (5)	
C12	0.7185 (5)	0.3371 (3)	0.70810 (17)	0.0378 (8)	
H12A	0.6999	0.4189	0.6915	0.045*	
H12B	0.8491	0.3184	0.7123	0.045*	
H12C	0.6709	0.3312	0.7498	0.045*	
C13	0.6627 (6)	0.0977 (4)	0.6968 (2)	0.0435 (9)	
H13A	0.6077	0.0285	0.6736	0.052*	
H13B	0.6187	0.1013	0.7392	0.052*	
H13C	0.7958	0.0893	0.7011	0.052*	

### Atomic displacement parameters (Å^2^)

**Table:**

	*U*^11^	*U*^22^	*U*^33^	*U*^12^	*U*^13^	*U*^23^
Ag1	0.02007 (14)	0.02377 (15)	0.02746 (16)	0.00319 (8)	0.00743 (10)	−0.00257 (9)
N1	0.0173 (12)	0.0213 (12)	0.0218 (13)	0.0015 (9)	0.0038 (9)	0.0011 (10)
N2	0.0171 (12)	0.0227 (12)	0.0201 (12)	0.0000 (9)	0.0017 (9)	−0.0040 (10)
N3	0.0251 (13)	0.0213 (13)	0.0214 (13)	0.0007 (10)	0.0055 (10)	−0.0009 (10)
C1	0.0199 (14)	0.0255 (15)	0.0236 (15)	−0.0021 (12)	0.0026 (11)	0.0025 (12)
C2	0.0298 (17)	0.0243 (15)	0.0261 (16)	0.0005 (13)	0.0006 (13)	−0.0054 (13)
C3	0.0281 (17)	0.0325 (18)	0.0313 (17)	0.0059 (13)	0.0056 (13)	−0.0069 (14)
C4	0.0165 (14)	0.0332 (17)	0.0375 (18)	0.0030 (12)	0.0069 (13)	−0.0017 (15)
C5	0.0169 (13)	0.0212 (14)	0.0221 (15)	0.0008 (11)	0.0013 (11)	0.0061 (12)
C6	0.0170 (14)	0.0236 (15)	0.0310 (17)	0.0014 (11)	0.0010 (12)	−0.0006 (13)
C7	0.0134 (13)	0.0206 (14)	0.0216 (14)	−0.0032 (10)	0.0004 (10)	−0.0035 (11)
C8	0.0237 (15)	0.0227 (15)	0.0237 (15)	−0.0005 (12)	0.0064 (12)	−0.0050 (12)
C9	0.0332 (18)	0.0316 (17)	0.0187 (14)	0.0039 (13)	0.0077 (12)	−0.0018 (13)
C10	0.0292 (16)	0.0281 (16)	0.0221 (15)	0.0026 (13)	0.0016 (12)	0.0026 (13)
C11	0.0145 (13)	0.0238 (14)	0.0183 (14)	−0.0020 (11)	0.0041 (10)	−0.0031 (11)
Cl1	0.0197 (4)	0.0316 (4)	0.0305 (4)	−0.0007 (3)	0.0042 (3)	0.0034 (3)
O1	0.127 (3)	0.064 (2)	0.0482 (19)	−0.056 (2)	0.023 (2)	−0.0070 (17)
O2	0.0408 (15)	0.0491 (16)	0.0449 (16)	−0.0090 (12)	0.0067 (12)	−0.0133 (13)
O3	0.067 (2)	0.082 (3)	0.055 (2)	0.0250 (19)	−0.0210 (16)	0.0001 (18)
O4	0.0364 (18)	0.107 (3)	0.073 (3)	0.0121 (17)	0.0135 (17)	−0.029 (2)
S1	0.0257 (4)	0.0319 (4)	0.0210 (4)	0.0002 (3)	0.0030 (3)	−0.0023 (3)
O5	0.0330 (13)	0.0431 (14)	0.0235 (12)	0.0003 (10)	0.0071 (10)	−0.0045 (10)
C12	0.046 (2)	0.041 (2)	0.0263 (17)	−0.0081 (17)	0.0023 (15)	−0.0101 (15)
C13	0.051 (2)	0.036 (2)	0.043 (2)	−0.0006 (17)	0.0024 (18)	0.0077 (17)

### Geometric parameters (Å, º)

**Table:**

Ag1—N2^i^	2.181 (2)	C7—C8	1.392 (4)
Ag1—N1	2.208 (2)	C7—C11	1.402 (4)
N1—C1	1.345 (4)	C8—C9	1.385 (4)
N1—C5	1.352 (4)	C8—H8	0.9500
N2—C11	1.332 (4)	C9—C10	1.384 (4)
N2—C10	1.346 (4)	C9—H9	0.9500
N2—Ag1^i^	2.181 (2)	C10—H10	0.9500
N3—C7	1.384 (4)	C11—H11	0.9500
N3—C6	1.460 (4)	Cl1—O4	1.412 (3)
N3—H3	0.8800	Cl1—O1	1.413 (3)
C1—C2	1.379 (4)	Cl1—O3	1.416 (3)
C1—H1	0.9500	Cl1—O2	1.457 (3)
C2—C3	1.385 (5)	S1—O5	1.515 (3)
C2—H2	0.9500	S1—C13	1.784 (4)
C3—C4	1.377 (5)	S1—C12	1.791 (4)
C3—H3A	0.9500	C12—H12A	0.9800
C4—C5	1.389 (4)	C12—H12B	0.9800
C4—H4	0.9500	C12—H12C	0.9800
C5—C6	1.506 (4)	C13—H13A	0.9800
C6—H6A	0.9900	C13—H13B	0.9800
C6—H6B	0.9900	C13—H13C	0.9800
			
N2^i^—Ag1—N1	170.78 (9)	C9—C8—C7	118.9 (3)
C1—N1—C5	118.0 (3)	C9—C8—H8	120.5
C1—N1—Ag1	118.47 (19)	C7—C8—H8	120.5
C5—N1—Ag1	121.8 (2)	C10—C9—C8	120.1 (3)
C11—N2—C10	118.6 (3)	C10—C9—H9	120.0
C11—N2—Ag1^i^	115.99 (18)	C8—C9—H9	120.0
C10—N2—Ag1^i^	124.9 (2)	N2—C10—C9	121.5 (3)
C7—N3—C6	121.0 (3)	N2—C10—H10	119.3
C7—N3—H3	119.5	C9—C10—H10	119.3
C6—N3—H3	119.5	N2—C11—C7	123.6 (3)
N1—C1—C2	123.6 (3)	N2—C11—H11	118.2
N1—C1—H1	118.2	C7—C11—H11	118.2
C2—C1—H1	118.2	O4—Cl1—O1	111.7 (3)
C1—C2—C3	118.3 (3)	O4—Cl1—O3	110.0 (3)
C1—C2—H2	120.8	O1—Cl1—O3	109.7 (3)
C3—C2—H2	120.8	O4—Cl1—O2	108.8 (2)
C4—C3—C2	118.8 (3)	O1—Cl1—O2	108.34 (19)
C4—C3—H3A	120.6	O3—Cl1—O2	108.1 (2)
C2—C3—H3A	120.6	O5—S1—C13	105.98 (17)
C3—C4—C5	120.1 (3)	O5—S1—C12	105.90 (16)
C3—C4—H4	119.9	C13—S1—C12	98.13 (19)
C5—C4—H4	119.9	S1—C12—H12A	109.5
N1—C5—C4	121.2 (3)	S1—C12—H12B	109.5
N1—C5—C6	119.0 (3)	H12A—C12—H12B	109.5
C4—C5—C6	119.8 (3)	S1—C12—H12C	109.5
N3—C6—C5	114.6 (2)	H12A—C12—H12C	109.5
N3—C6—H6A	108.6	H12B—C12—H12C	109.5
C5—C6—H6A	108.6	S1—C13—H13A	109.5
N3—C6—H6B	108.6	S1—C13—H13B	109.5
C5—C6—H6B	108.6	H13A—C13—H13B	109.5
H6A—C6—H6B	107.6	S1—C13—H13C	109.5
N3—C7—C8	124.0 (3)	H13A—C13—H13C	109.5
N3—C7—C11	118.6 (3)	H13B—C13—H13C	109.5
C8—C7—C11	117.3 (3)		
			
C5—N1—C1—C2	0.2 (4)	C4—C5—C6—N3	−143.6 (3)
Ag1—N1—C1—C2	−165.1 (2)	C6—N3—C7—C8	−9.0 (4)
N1—C1—C2—C3	0.5 (5)	C6—N3—C7—C11	173.9 (3)
C1—C2—C3—C4	−0.9 (5)	N3—C7—C8—C9	−177.2 (3)
C2—C3—C4—C5	0.7 (5)	C11—C7—C8—C9	0.0 (4)
C1—N1—C5—C4	−0.4 (4)	C7—C8—C9—C10	0.8 (5)
Ag1—N1—C5—C4	164.3 (2)	C11—N2—C10—C9	−0.3 (4)
C1—N1—C5—C6	177.6 (3)	Ag1^i^—N2—C10—C9	170.8 (2)
Ag1—N1—C5—C6	−17.7 (4)	C8—C9—C10—N2	−0.7 (5)
C3—C4—C5—N1	0.0 (5)	C10—N2—C11—C7	1.2 (4)
C3—C4—C5—C6	−178.0 (3)	Ag1^i^—N2—C11—C7	−170.7 (2)
C7—N3—C6—C5	75.8 (3)	N3—C7—C11—N2	176.4 (3)
N1—C5—C6—N3	38.4 (4)	C8—C7—C11—N2	−1.0 (4)

Symmetry code: (i) −*x*+1, −*y*, −*z*+1.

### Hydrogen-bond geometry (Å, º)

Cg is the centroid of the N2/C7–C11 pyridine ring.

**Table:**

*D*—H···*A*	*D*—H	H···*A*	*D*···*A*	*D*—H···*A*
N3—H3···O1	0.88	2.32	3.081 (4)	144
C1—H1···O2^ii^	0.95	2.56	3.214 (4)	126
C6—H6*A*···O5^iii^	0.99	2.55	3.495 (4)	160
C11—H11···O5^i^	0.95	2.55	3.191 (4)	125
C12—H12*C*···O4^iv^	0.98	2.49	3.270 (5)	137
C13—H13*A*···*Cg*	0.98	3.36	4.116 (5)	136

Symmetry codes: (i) −*x*+1, −*y*, −*z*+1; (ii) −*x*+1, −*y*+1, −*z*+1; (iii) *x*−1, *y*, *z*; (iv) *x*, −*y*+1/2, *z*+1/2.
